# Strength and Leaching Behavior of Contaminated Mining Sludge at High Water Content Stabilized with Lime Activated GGBS

**DOI:** 10.3390/ma14216524

**Published:** 2021-10-29

**Authors:** Traore Abdoul Fatah, Rongjun Zhang, Xiaosong Huang, Junjie Zheng, Yu Miao, Aamir Khan Mastoi

**Affiliations:** Institute of Geotechnical and Underground Engineering, Huazhong University of Science and Technology, Wuhan 430074, China; abdoulfatah@hust.edu.cn (T.A.F.); ce_zhangrj@hust.edu.cn (R.Z.); zhengjj@hust.edu.cn (J.Z.); miaoyu@hust.edu.cn (Y.M.); ak.mastoi21@gmail.com (A.K.M.)

**Keywords:** mining sludge, solidification/stabilization, cement, ground granulated blast furnace slag, strength, heavy metal leachability

## Abstract

Sludge management is one of the major challenges in mining activities. The direct disposal of contaminated mining sludge can bring severe damages to the environment and community. Solidification/stabilization (S/S) is a very efficient technology for the treatment of contaminated mining sludge because it improves the stability of sludge dumping sites and reduces the leachability of contaminants. Very few studies investigate the S/S of mining sludge, especially with high water content. This paper investigated the effectiveness of S/S for the treatment of mining sludge at high water content by using quick lime (CaO) activated ground granulated blast furnace slag (GGBS) in comparison to ordinary Portland cement (OPC). To evaluate the mechanical, leaching, and microstructural behavior of CMS at high water content stabilized by lime-activated GGBS and OPC, a series of laboratory experimental tests were performed. Experimental results indicated that increasing the dosage of binder led to increased strength and decreased leachability of the heavy metal. In contrast, an increase in the water content of the mixture resulted in a decrease in compressive strength and an increase in the leachability of heavy metals. On the other hand, lime-activated GGBS mixes had substantially better performance than OPC mixes in the aspect of strength development of treated mining sludge and showed comparable capability of heavy metal stabilization compared to OPC. The microstructural tests revealed the formation of different hydration products such as calcium silicate hydrate, calcium aluminum silicate hydrate, ettringite, hydrotalcite, and heavy metal complexes in CG and OPC mixes.

## 1. Introduction

Mining activities are essential in the economic development of many countries over the world. The extraction of minerals presents opportunities, challenges, and risks to sustainable development. Mining exploitation often leads to environmental and ecological challenges, such as soil and underwater pollution in mining areas, plant destruction and biodiversity loss, and geological and land destruction. The most widely used contaminated mining sludge disposal method is pond disposal. However, the disposal of heavy metal contaminated mining sludge (CMS) at high-water content could cause significant environmental and ecological damages [1]. Furthermore, the tailings dam collapse caused environmental and natural disasters that can have severe consequences of loss of life, environmental and financial consequences in billions of dollars [2]. Therefore, the effective remediation of a high water content CMS has drawn the interest of researchers worldwide.

There are many remediation techniques available to treat such high water content CMS to avoid environmental pollution. However, the Solidification/stabilization (S/S) treatment method is attractive for many wastes, including CMS, by utilizing cement, lime, and other binders to minimize the toxicity of contaminants and enhance mechanical strength before final disposal [3]. Solidification/stabilization is one of the most applied technology to improve sludge stability [4] and is a very recognized technique for the treatment of heavy metal contaminated soils [5]. After mixing the binder with the sludge, the binders react with heavy metal salts and form precipitations (i.e., compounds or insoluble complex hydroxides) due to their alkaline nature [6]. Furthermore, the heavy metals are encapsulated by hydration products such as calcium silicate hydrate (CSH), calcium aluminate hydrate (CAH) generated during the hydration process [7].

However, because the manufacturing of cement and lime is very often associated with enormous energy consumption and generates very high carbon dioxide emissions in the environment [8], researchers have recently been attempting to develop an eco-friendly alternative for these conventional binders in recent years with the intention of using industrial waste such as ground granulated blast furnace slag (GGBS). GGBS is a by-product of the steel industry, and its production requires low energy consumption and carbon emission [9]. It is an eco-friendly binder for engineering projects and can substitute or partially replace cement or lime in soil treatment [10]. Several researchers studied the solidification/stabilization of heavy metal contaminated soils using GGBS [11]. The results suggested that activated GGBS could effectively improve the mechanical properties of contaminated soils and avoid the leaching of the contaminant into the environment [12].

Nevertheless, without an activator, GGBS cannot completely react with the soil particles. In fact, in the S/S process, the strength of the matrices containing only GGBS is generally lower than that of the samples containing activated GGBS, which indicates that the presence of an activator can considerably improve the mechanical behavior of the GGBS system [13] and reduce the leachability of heavy metal. The type of activator has a considerable impact on the resistance of treated soils [14]. Generally, cement and lime are common activators used in the GGBS system [15]. In the case of GGBS, calcium hydroxide Ca(OH)_2_ and calcium oxide (CaO) are effective activators because they are readily available and significantly less expensive than other activators such as OPC, sodium silicate, and sodium hydroxide [16]. Comparing the effect of CaO and Ca (OH)_2_ on GGBS activation revealed that CaO had a greater mechanical strength and activation potential for GGBS than Ca(OH)_2_ [17]. Some recent studies also demonstrated that GGBS activated by reactive magnesia (MgO) with a perfect ratio led to a higher UCS than OPC or GGBS-CaO blends [18]. However, the application of MgO-GGBS in soil treatment remains limited because the cost of magnesia-activated GGBS in the treatment of soil is more expensive than lime [16]. Using lime as an activator of GGBS in soil treatment can be a way to cut down the total cost of treatment.

To date, very few studies investigated the effects of lime-activated GGBS, on the solidification/stabilization process of mining sludge, especially with high water content. This study aims to gain an insight into the strength characteristics and heavy metal leaching behavior of contaminated mining sludge at high water content solidified/stabilized with lime-activated GGBS or OPC. It considers the leachability of three contaminants, Cu, Pb, and Zn, which are among the commonly encountered heavy metals in the soil [19]. In fact, Cu, Pb, and Zn rank amongst the fifth heavy metal in the industrial production of metal [20], and they represent the most common heavy metals found at contaminated sites [21]. Pb, Cu, and Zn are significant because they have the potential to reduce crop output due to bioaccumulation and biomagnification in the food chain, the possibility of contamination of soil, groundwater, vegetation, and air pollution, and resulting ecological environmental diseases during the processing of mining operations [22].

Microstructural characteristics were also investigated through X-ray diffraction (XRD) and scanning electron microscopy (SEM) to better understand the change in strength (constitution).

## 2. Material and Method

### 2.1. Materials

**Figure 1:**
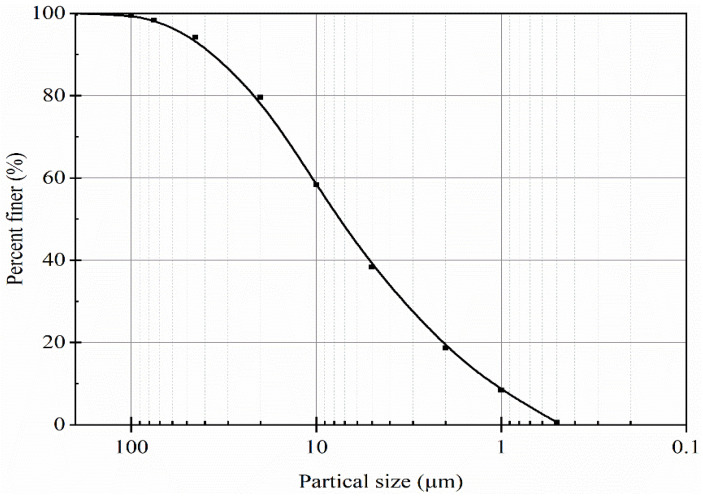
Particle size distribution of the sludge used in the laboratory experiments.

Contaminated sludge used in the laboratory experiments was collected from an actual copper mine site. As summarized in Table 1, basic physiochemical characteristics of the used sludge were determined according to China Standard GB/T 50123-2019, “Standard for geotechnical testing”. The particle size distribution is shown in Figure 1, and concentrations of heavy metals (Cu, Pb, and Zn) were tested using flame atomic absorption spectrometry and presented in Table 1.

In this study, ground granulated blast furnace slag (GGBS) was selected because of its environmental, technical, and economic benefits [15,23]. As recently mentioned above, reactive magnesia is proved to be a good activator for GGBS. However, the price is costly. In China, for example, the price of magnesia varies from US$180 to US$350 per ton compared to lime (i.e., US$30 to US$80 per ton) according to Beijing HL Consulting Company 2009 [24]. In this study, using lime as an activator of GGBS in the stabilization of contaminated CMS is a way to cut down the total cost of treatment. Another reason for selecting lime is its high efficiency for heavy metal precipitation. The ordinary Portland cement was also used as the conventional binder in solidification/stabilization for comparison. The OPC used in this experiment was OPC.42.5 and is manufactured in China. The GGBS and CaO used for the experiment were obtained as a white powder from a local supplier in Wuhan. The physicochemical properties of GGBS, CaO, and OPC were determined via X-ray fluorescence (XRF) test and listed in Table 2. As previously mentioned, heavy metals such as Cu, Pb, and Zn were targeted. Finally, Zn(NO_3_)_2_·6H_2_O, Pb(NO_3_)_2_, and Cu (NO_3_)_2_·3H_2_O were chosen to prepare the contaminated sludge and were obtained from Wuhan Xinshenshi Chemical Technology Co., Ltd. The reason for using nitrate as the contamination source is that nitrate is inert to cement hydration [25].

### 2.2. Preparation of Contaminated Mining Sludge (CMS) at High Water Content

Testing materials were obtained from real tailings with their original amount of contaminants, as shown in Table 1 would have another added value for the mining company. However, this current study investigates the effects of lime-activated GGBS and ordinary Portland cement-treated high concentrations of several heavy metals. In addition, the initial total heavy metal concentrations showed in Table 1 were below the standard value according to the background value of soil environment in China (China National Environmental Monitoring Center, Beijing, China, 1990). Furthermore, one contaminant concentrations (i.e., (i) Cu. 2901.53 mg/kg, (ii) Pb. 94.38 mg/kg, and (iii) Zn. 1614.73 mg/kg), corresponding to middle pollution of Pb and the high concentration degree of Zn were selected as the target values for Cu, Pb, and Zn, contaminated CMS in this study according to the background value of soil environment in China [26]. Four water contents varying from 100% to 160% have been considered in the experiments. Moreover, contaminated sludge specimens were prepared by dissolving the predetermined amount of Zn (NO_3_)_2_·6H_2_O, Pb (NO_3_)_2_, and Cu (NO_3_)_2_·3H_2_O solution in water and mixing with the sludge. The mixing was carried out through an electric agitator for 10 min following the standardized mixing procedure and braised for 10 days under standard curing conditions to allow heavy metal and sludge to reach equilibrium [25]. The binder content was set at 10%, 12%, 15%, and 20% by weight of dry sludge weight, respectively. In the case of lime-activated GGBS, the quicklime (the activator) to GGBS ratio is 1:3, as recommended by [5,12]. Furthermore, the binders were added to the contaminated sludge on predetermined dry sludge weight and mixed thoroughly for 10 min with an electronic mixer to obtain a homogenous mixture. The mix was then poured in cylindrical molds (50 mm in diameter and 100 mm high) and were cured in the curing box at temperatures 25 ± 1 °C and humidity was maintained at 95 ± 3%.

### 2.3. Testing Procedure

A total of 16 cases were conducted during the laboratory experiment, and the designed proportions are summarized in Table 3. The 16 cases were divided into four groups (i.e., A–D). Group A includes four CMS cases stabilized with different values of OPC content. Group B includes four CMS cases stabilized with OPC at different values of water content. Furthermore, Group C and D include four CMS cases stabilized with lime-activated GGBS at different binder content and different water content.

Four curing times were considered for each testing case (7, 14, 21, and 28 days). In fact, the strength of a soil–cement composite improves with curing age. However, preliminary research was conducted in this study to compare the efficacy of S/S for the treatment of mining sludge with high water content utilizing quick lime (CaO) activated ground granulated blast furnace slag (GGBS) to ordinary Portland cement (OPC). After 7, 14, 21, and 28 days, specimens were extruded from the molds, and unconfined compression strength tests (UCS) were conducted to determine their crushing strength according to the ASTM standard D-1633. Following UCS, the samples were crushed to reduce the particle size to less than 2 mm in order to determine the specimen’s leachability using the toxicity characteristics leaching procedure experiment (TCLP) defined by EPA method 1311. For XRD analysis, the samples obtained from UCS were crushed and sieved through a 0.075 mm sieve to get a fine powder, and the samples were scanned in ranges from 10 to 70 (2∅) using a Rigku D/Max-2500 X-ray diffractometer with a Cu-Kα source to identify the crystalline phases. Scanning electron microscopy (SEM) was also used on the selected samples to analyze the microstructure properties of the stabilized soils.

## 3. Results and Discussion

### 3.1. Strength Characteristics of Treated Mining Sludge at High Water Content

**Figure 2:**
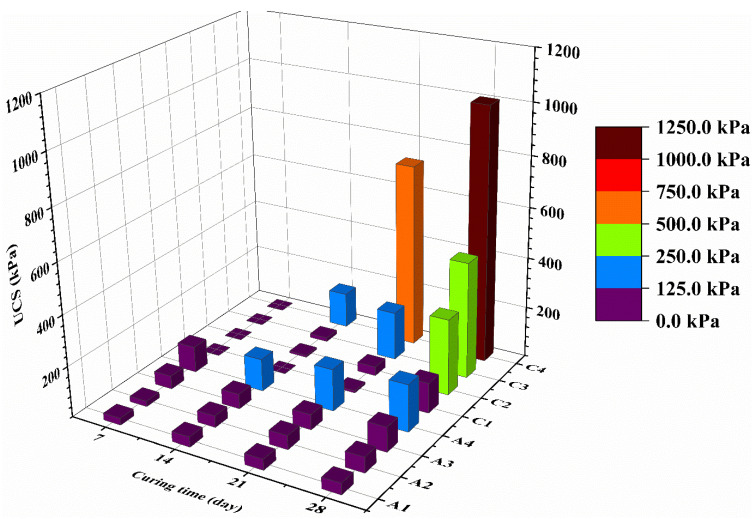
UCS of solidified samples with the same water content.

Figure 2 illustrates the UCS results of CMS at high water content stabilized by CG and OPC at different binder content and curing time (groups A and C). The CG stabilized CMS samples were not strong enough to be de-molded after 7-day of curing time. The CG stabilized CMS showed a lower 7-day UCS than OPC stabilized CMG, but the formers produced higher UCS values at later curing ages. This is attributed to the slow hydration rate of GGBS at an early age, which has nevertheless resulted in higher long-term strength once activated, as reported in previous studies [27,28]. The 28 days UCS of the stabilized CMS specimens increases with binder content and curing time as expected due to the formation of cementitious phase. When the CG content increased from 10 to 20%, the compressive strength increased to 118.18 kPa and 1003.86 kPa at 28-day curing time, respectively.

Furthermore, the UCS values of CG stabilized CMS increased significantly with curing time compared to OPC stabilized CMS. After 28-day of curing time, CG stabilized CMS showed 5.44 times higher UCS than OPC stabilized CMS at the same water content and binder content. This could be explained by the higher hydration rate of GGBS activated by lime and the increase of hydrates, such as CSH and CASH, hydrotalcite-like phases in stabilized CMS, which can increase the UCS of samples. None of the OPC stabilized CMS fulfilled the US EPA criterion (0.35 MPa) even after the 28-day curing time due to their lower strength, whereas the CG stabilized CMS met these criteria after the 21-day curing time.

**Figure 3:**
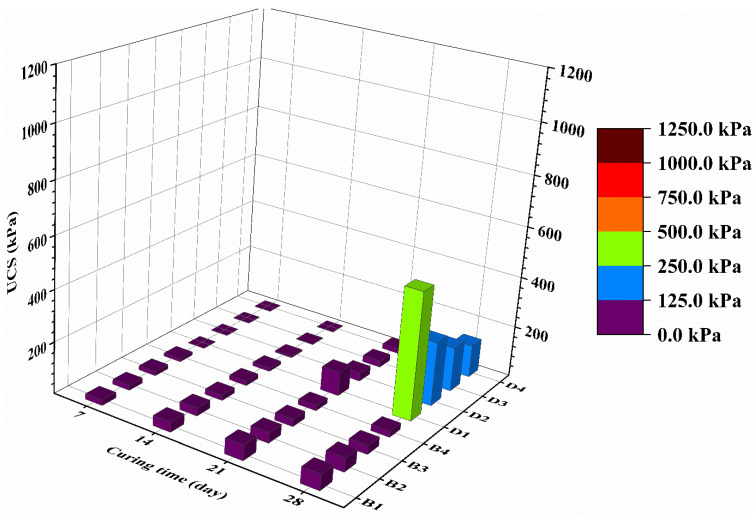
UCS of solidified samples at the same binder content and different water content.

Figure 3 showed the 28-day UCS results of stabilized CMS at different water content values (cases B and D). It can be observed that, at 28-day, the UCS decreases with the increase of water content for both stabilized specimens. Indeed, the UCS decreases approximately by 3.9 times for CG stabilized specimens and 1.7 times for OPC samples when increasing the water content from 100 to 160% after 28-day of curing. The strength of a stabilized soil also depends on the water content of the soil–cement mixture, as it is for the concrete mixture. It is known that UCS depends on the quality of the pore structure of cement stabilized matrix. Additionally, this quality is influenced by the type and quantity of constituents that contribute to the pore structure, namely OPC hydration products. However, for all the stabilized CMS, the CG produces a higher UCS up to eight orders of magnitude than OPC stabilized samples. These results were attributed to the formation of a more voluminous hydration product such as hydrotalcite (Ht) in the CG stabilized CMS [29]. Ht is more voluminous than C S H, which effectively fills soil pores and leads to higher strength developments [18,30]. Additionally, stabilized high water content CMS can be used as structural backfill material because the minimum strength required is 100 KPa, as reported by [31].

### 3.2. Heavy Metal Leaching Behavior of Treated Mining Sludge at High Water Content

**Figure 4:**
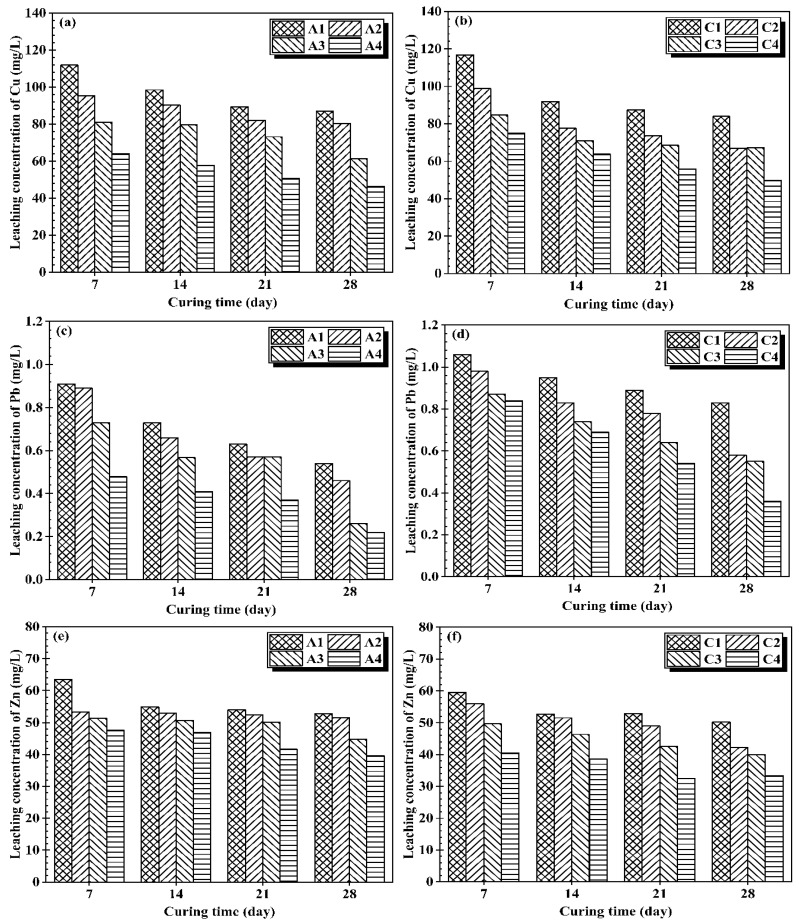
Leaching concentration vs. binder percentage: (**a**) Cu in OPC samples, (**b**) Cu in CG samples, (**c**) Pb in OPC samples, (**d**) Pb in CG samples, (**e**) Zn in OPC samples, (**f**) Zn in CG samples.

The TCLP experiments were also carried out on stabilized CMS samples, which could represent the long-term stability of S/S material in the context of leaching. Figure 4 shows the detailed results of heavy metal leachability of Cu, Pb, and Zn stabilized by CG and OPC at the same water content and different binder content. It can be seen that leaching concentration of heavy metals such as Cu, Pb, and Zn from stabilized CMS were lower than 100 mg/L, 5 mg/L, and 100 mg/L, respectively, which are the regulatory limit specified by Chinese standard method. The leachability of heavy metals decreases with the curing time and the increase of binder content for both CG and OPC stabilized CMS. This shows that by incorporating binders, the leachability of heavy metals in CMS decreases, which is primarily due to their insoluble hydroxides and/or complexes, as seen in the XRD result (Figure 6).

The leached Cu and Pb concentrations from OPC stabilized CMS were on the lower side than CG stabilized CMS. More specifically, OPC stabilized CMS exhibited 6.95% and 38.8% lower leached concentration of Cu and Pb than CG cases, respectively. The improved immobilization capability of OPC is attributed to encapsulation within the OPC structure, as well as the chemical reaction between Ca(OH)_2_ and heavy metals [32]. This is consistent with the findings of [33], who revealed that OPC-treated contaminated soil had lower Pb and Cu leachability than CaO-treated contaminated soil. In contrast, CG stabilized CMS showed 15.7% lower leachability of Zn than OPC stabilized CMS.

This pronounced decrease in Zn leachability in CG cases is due to the production of more voluminous hydration products such as CSH and hydrotalcite, resulting in a dense stabilized matrix that provides greater resistance to TCLP acid solution [34]. Besides, hydrotalcite formed in CG cases (see XRD results) is an effective heavy metal absorber and reduces Zn’s leachability through isomorphic substitution [18,35].

**Figure 5:**
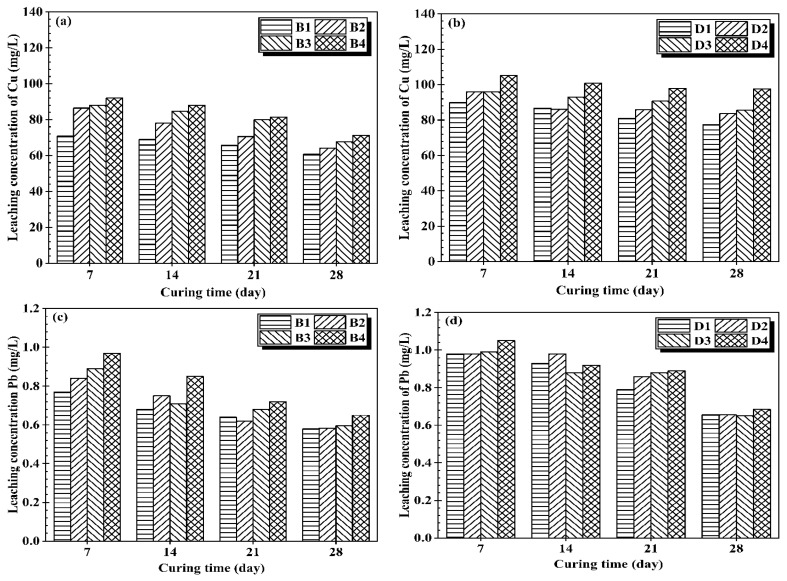

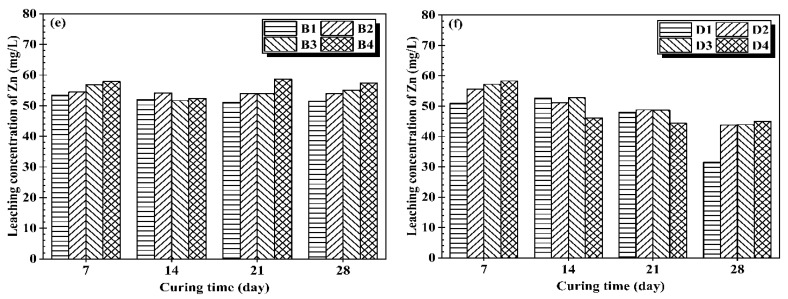
Leaching concentration vs. water content: (**a**) leachate concentration of Cu in OPC samples, (**b**) leachate concentration of Cu in CG samples, (**c**) leachate concentration of Pb in OPC samples, (**d**) leachate concentration of Pb in CG samples, (**e**) leachate concentration of Zn in OPC samples, (**f**) leachate concentration of Zn in CG samples.

The increase in water content (Figure 5) led to the rise in the leachability of heavy metals in both cases. The leachability increases significantly when increasing the water content from 100% to 160%. For all cases, OPC stabilized CMS leach out a higher concentration of Zn than CG stabilized CMS except for Pb and Cu. Analysis of Figure 3 and Figure 5 showed that the treated CMS with a high water content present a lower UCS and higher leachability compared to those treated with lower water content. Furthermore, the CMS stabilized by CG showed better performance than OPC treated specimens, which could be responsible for better encapsulation of heavy metal [30]. Although the increase of water content has a significant effect on OPC stabilized CMS, the heavy metal concentrations in the leachate were below the regulatory limit according to the Chinese standard after 28 days of curing. The increase in water content did not significantly affect the immobilization of Pb because the initial concentration of Pb on the CMS is not that higher. Therefore, CG-based solidification/stabilization can be used for the safe disposal of high water CMS treated at 12% binder content. The replacement of OPC with lime-activated GGBS lead to an improvement in the heavy metal retention compared to OPC stabilized CMS. The above findings demonstrated that the proposed CG binder was effective in the S/S of heavy metal contaminated sludge at high water content.

### 3.3. XRD Analysis of Treated Mining Sludge at High Water Content

**Figure 6:**
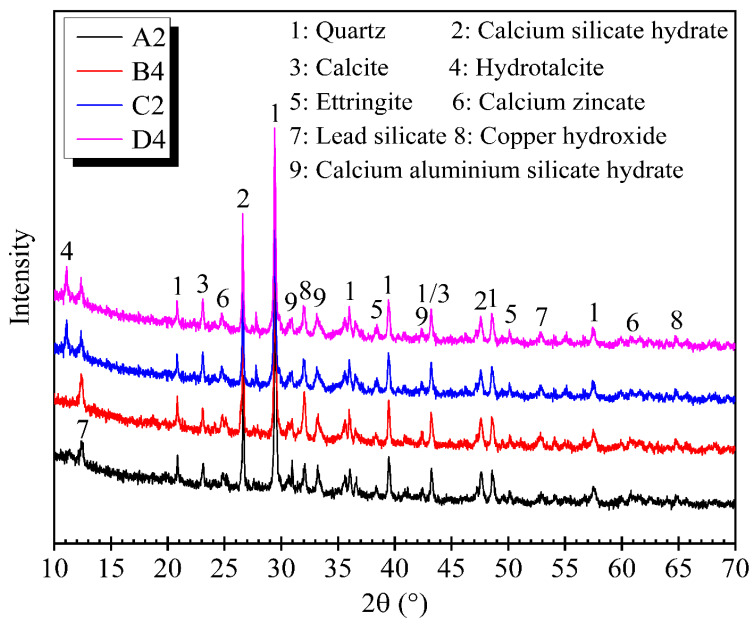
XRD diffractograms of CMS samples treated by OPC and CG after 28 days of curing.

The 28-day crystalline phases of OPC and CG cases determined by XRD analysis are shown in Figure 6 Quartz has been found as the common compound of CMS, reflecting the nature of used mining sludge. Typical hydration products such as Calcium silicate hydrate (CSH), calcium aluminate silicate hydrate (CASH), and ettringite were also identified in both OPC and CG cases, suggesting that the major hydration products of CG stabilized CMS were similar to that of OPC stabilized CMS. This is in agreement with previous findings [16,36]. However, the additional peaks of hydrotalcite were also detected in CG cases, which is the only difference between the hydration products of CG and OPC stabilized CMS. Hydrotalcite formation in the CG system is expected as a result of magnesium dissolution and precipitation from the GGBS particles. The development of these voluminous hydration products could increase the binding capability, resulting in higher strength development of stabilized CMS [12]. Calcite was also detected, which is the result of the reaction between CaO and gas-phase CO_2_.

Under high alkaline conditions, Pb was solidified/stabilized on the surface of CSH by an adsorption mechanism and chemical reactions to form insoluble lead silicate, as shown in Figure 6 Trace peaks of Zinc oxide and copper oxide were identified in both specimens (Figure 6), indicating that Cu and Zn were mainly precipitated as oxide. Zinc silicate has also been identified in XRD patterns of both cases, which is parallel with the findings of [37], who reported that Zn is usually bound to carbonate and Fe/Mn oxide phases. The Zn tetrahedral can also be bound to the CSH tetrahedral silicate chains, leading to Zn retention. Zn was then stabilized/solidified by CSH adsorption, precipitation, and incorporation into the components of hydration products such as CSH and hydrotalcite. Another complex called calcium zincate (CaZn_2_ (OH) 6·2H_2_O) was also observed in both cases, which is supposed to form from the Ca(OH)_2_ and Zn(OH)_2_ reaction. In addition, due to Zn’s retardant effect on cement hydration, portlandite (Ca(OH)_2_,) one of the major hydration products, was not detected in the OPC stabilized CMS, which is consistent with a finding previously reported in the literature [25]. No portlandite was also detected in the CG cases. The absence of portlandite in the CG stabilized samples is due to its consumption during the GGBS activation, which agrees with [38].

### 3.4. SEM Images for Treated Mining Sludge at High Water Content

**Figure 7:**
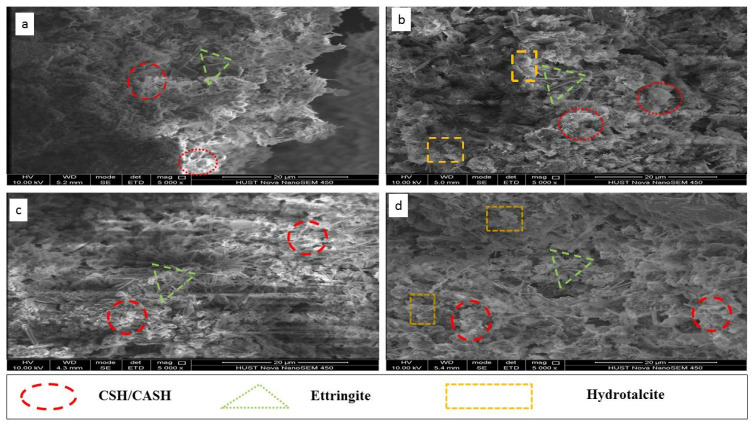
Microstructure of contaminated sludge treated by OPC and CG after 28 days of curing: (**a**) case A2, (**b**) case C2, (**c**) case B4, (**d**) case D4.

SEM tests were performed to examine the microstructure development on the typical 28-day OPC and CG stabilized CMS specimens, and the results are shown in Figure 7. The OPC stabilized CMS microstructure with 12% OPC at 120% water content is shown in Figure 7a, and the CG stabilized CMS with 12% CG at 120% water content is shown in Figure 7b. The analysis of the micrograph indicates that the soil particles were disorderly distributed with a large number of small pores. The CG and OPC hydration products of gel-like CSH, platy CASH gels, platelet hydrotalcite, and needle-like ettringite crystal have been filled into the pores of these CMS particles, leading to the disappearance of large-scale pores. This is consistent with [39], who reported that CASH appeared to be platy in soil–lime/cement reaction. Such cementation and filling ability of hydration products contributed to the strength development of stabilized CMS. The analysis of Figure 7c,d showed a large amount of pore, indicating that the increase of water content from 120% to 160% for OPC and CG treated specimens significantly affected the microstructure.

When increasing the water content, the hydrates products in both cases remain the same such as CSH, CASH, hydroltalcite, and ettringite. However, a large quantity of pores has been detected (Figure 7c,d). Indeed, the microstructure of the stabilized CMS particles changed from a dense structure to a dispersed nature with a large number of pores due to the presence of a large amount of water. The authors of [40] previously reported similar observations due to an increase in water content. Figure 7b,d depict the microstructure of CG stabilized CMS samples with a 12% binder content produced, and the CMS particles have been strongly cemented, resulting in significant improvement in the development of strength. This agrees with the UCS results presented in Figure 3 and Figure 4 showing that the 28 days UCS of CMS stabilized with CG were higher than OPC under the same water content.

## 4. Conclusions

In this study, lime-activated GGBS based solidification/stabilization has been proposed for the treatment of high water CMS. A series of tests were conducted to evaluate the effect of water content and binder content on the strength characteristics and leaching behavior of the treated material. The main conclusions drawn from the analysis include:

1. Lime-activated GGBS has substantially better performance than OPC in the aspect of strength development of treated mining sludge. At 28-day, the UCS of CG stabilized CMS showed 5.44 times higher UCS than OPC stabilized CMS at the same water content and binder content.
2. Both CG and OPC samples exhibit a decrease in the leaching concentration of heavy metal with an increase in curing time. However, CG stabilized samples show comparable capability of heavy metal stabilization in contrast to OPC.
3. XRD patterns showed that the main hydration products of both CG and OPC mixes were CSH, CASH, and ettringite. The hydrotalcite produced in the CG mix was the only difference between the hydration products of CG and OPC mixes.
4. SEM micrographs exhibited that CG mix developed dense microstructure due to the formation of more voluminous hydration products such as hydrotalcite, filling the pores between CMS particles more effectively, resulting in a dense stabilized matrix.

## Figures and Tables

**Table 1:** Physical properties of the mining sludge collected from the actual copper mine site.

Property		Value
Nature moisture content, %		50
pH		7.8
Specific gravity		2.61
Liquid limit LL, %		41.45
Plastic limit PL, %		24.43
Plasticity Index		17.02
Particle Size Distribution	Sand fraction (0.075–2 mm), %	1.68
	Silt fraction (0.002–0.075 mm), %	79.74
	Clay and colloid fraction (<0.002 mm), %	18.58
Soil Classification		Lean clay CL
Total Cu concentration, mg/kg		609.92
Total Pb concentration, mg/kg		15.6
Total Zn concentration, mg/kg		274.9

**Table 2:** Chemical compositions of materials.

Composition	CaO (wt.%)	SiO_2_ (wt.%)	Al_2_O_3_ (wt.%)	Fe_2_O_3_ (wt.%)	MgO (wt.%)	K_2_O (wt.%)	SO_3_ (wt.%)	Na_2_O (wt.%)	Others (wt.%)	Loss on Ignition (wt.%)
OPC	59.81	22.33	6.26	2.54	3.41	0.70	4.02	0.68	-	0.25
CaO	92.1	1.46	0.68	0.101	4.79	0.017	0.19	-	0.152	-
GGBS	38.00	36.3	14.29	0.24	7.74	0.43	2.33	0.22	-	0.45
CMS	48.38	21.59	6.56	15.80	3.39	0.65	1.20	-	1.79	0.64

**Table 3:** Testing program.

Mix Type	Binder Type	Group	Case No.	*W* (%)	*A*_w_ (%)	Curing Time	No. of Specimens Prepared	Testing Items
OPC	Ordinary Portland cement	A	A1	120	10	7, 14, 21 and 28 days	8	UCS at 7,14, 21 and 28-day for all specimens TCLP at 7, 14, 21, and 28-day for all specimens XRD at 28-day for selected specimens SEM at 28-day for selected specimens
			A2	120	12		8	
			A3	120	15		8	
			A4	120	20		8	
		B	B1	100	12		8	
			B2	120	12		8	
			B3	140	12		8	
			B4	160	12		8	
CG	CaO-GGBS (1:3)	C	C1	120	10		8	
			C2	120	12		8	
			C3	120	15		8	
			C4	120	20		8	
		D	D1	100	12		8	
			D2	120	12		8	
			D3	140	12		8	
			D4	160	12		8	

## Data Availability

Data is contained within the article.

## Nomenclature

CMS	contaminated mining sludge
S/S	solidification/stabilization
U.C.S.	unconfined compressive strength
TCLP	toxicity characteristics leaching procedure
ASTM	American standard of testing material
EPA	environment protection agency
*W*	water content
*A*_w_	binder content
OPC	ordinary Portland cement
CG	lime activated GGBS
XRD	X-ray diffraction
XRF	X-ray fluorescence
SEM	scanning electron microscopy
Ht	hydrotalcite
